# How do core surface flow models vary when inverted from IGRF-14 candidate field models?

**DOI:** 10.1186/s40623-025-02307-5

**Published:** 2026-01-22

**Authors:** H. F. Rogers, M. Mandea

**Affiliations:** 1 School of Earth and Environment, University of Leeds, Woodhouse, Leeds, LS2 9JT UK; 2 Centre National d’Études Spatiales, 2 Place Maurice Quentin, 75039 Paris Cedex 01, France

**Keywords:** Core surface flow inversion, Secular variation, International Geomagnetic Reference Field (IGRF)

## Abstract

The International Geomagnetic Reference Field (IGRF) model is a series of models that describe the large scale magnetic field measured at the Earth’s surface. It is used by a wide range of scientists and industries, including in navigation, space weather applications, and resource exploration. The 14th generation, IGRF-14, is the result of an international collaboration over 19 different lead research groups. This new generation provides a definitive model of the main magnetic field (MF) for the epoch 2020.0, a prediction of the MF for 2025.0, and a predicted average annual time variation of the magnetic field for 2025.0$-$2030.0. The first time derivative of the magnetic field, known as secular variation, (SV) is linked to the flow at the top of Earth’s outer core. As such, the ensemble of IGRF-14 candidate models can be used to investigate predicted core flow variability between 2020.0 and 2030.0. We use the pygeodyn Python package using the AR-1 ‘dense’ methodology, the 71% geodynamo prior, and keep all inversion parameters fixed to ensure that differences in inferred flow arise solely from variations from the IGRF candidate models. Our results demonstrate that while all candidates produce broadly similar flow spectra for all degrees, candidates that deviate most from the median IGRF model also show the largest differences in flow. In all cases, the flow speed difference remains below 25% of the maximum flow speed of the Huber-weighted mean model in space, with most discrepancies occurring at small spatial scales. The greatest flow uncertainty appears over the Pacific and in the polar regions, where constraints are weaker. Finally, we assess the impact of the maximum spherical harmonic degree truncation of the SV candidate models by comparing two SV candidates truncated at spherical harmonic degree 13 instead of 8. Increasing the truncation degree of SV alters the flow spectral energy at all degrees and increases the maximum flow speed by up to 31%, despite the flow maps remaining highly correlated. This study supports the idea of raising the spherical harmonic truncation degree to 13 for the SV prediction component of the IGRF.

**Graphical Abstract:**

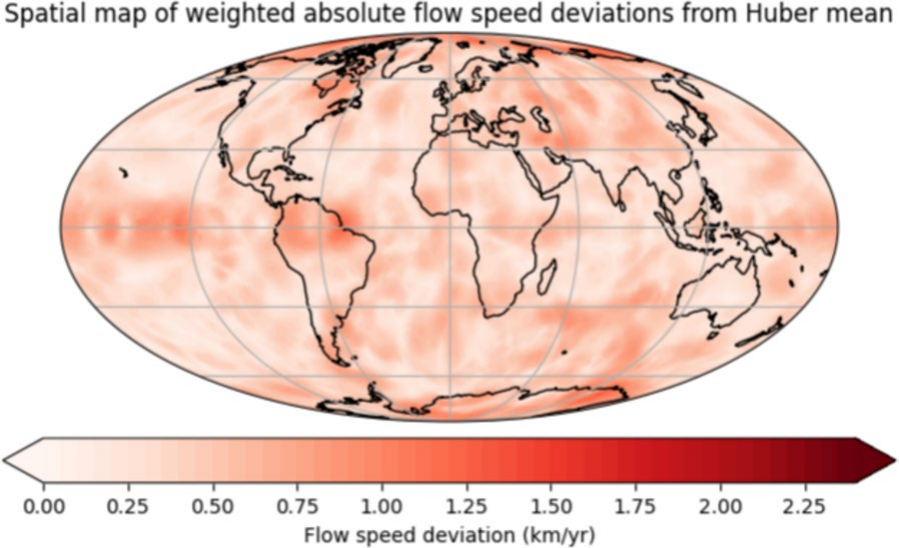

## Introduction

The Earth’s magnetic field is a constantly changing yet integral component of Earth’s ability to support life. Beyond serving as a protective shield against solar and cosmic radiation, many technological advancements are reliant on it, particularly navigation and orientation (Alken et al, 2021; Chulliat et al, 2020; Russell et al, 2016). The dominant contribution to the Earth’s magnetic field observed at the Earth’s surface is the core field, also named the main field (MF), which is generated by the convection of liquid metal in Earth’s outer core (Olsen et al. 2010). This ‘geodynamo’ process is inherently chaotic, arising from the interplay of thermal convection, planetary rotation, and compositional buoyancy. Due to computational constrains, numerical simulations cannot fully replicate the geodynamo under Earth-like conditions (Christensen and Wicht 2015; Jones 2011). Internally generated geomagnetic fields are typically represented by spherical harmonics defined in spherical coordinates ($r,\theta ,\phi$) at time *t* as

1 $$\begin{aligned} &  B_r(r,\theta ,\phi ,t) \nonumber \\ &  = \sum _{l=1}^L(l+1)\left( \frac{a}{r}\right) ^{l+2}\nonumber \\ & \quad \sum _{m=0}^l \left( g_l^m(t)\cos (m\phi ) +h_l^m(t)\sin (m\phi )\right) P_l^m(\cos \theta ), \end{aligned}$$

where *L* is the maximum spherical harmonic degree, *a* = 6371.2 km is the Earth’s reference radius, ${P}_{l}^{m}$ the associated Legendre polynomials of degree *l* and order *m*, and ($g_l^m,h_l^m$) the Schmidt semi-normalized Gauss coefficients (Gauss 1838; Goldie and Joyce 1940). Models of the first time-derivative, known as secular variation (SV), are represented in a similar way with SV Gauss coefficients $\dot{g}_l^m(t)$ and $\dot{h}_l^m(t)$.

The International Geomagnetic Reference Field (IGRF) model describes the global geomagnetic field at different epochs and is updated every 5 years to reflect the latest available observations (Alken et al. 2021; Finlay et al. 2010b; Macmillan 2003; Mandea and Macmillan 2000; Maus et al. 2005; Thébault et al. 2015). This model is widely used in various scientific and industrial applications, such as navigation, resource exploration, geophysics, and space weather studies, and has three components: (i) the ‘definitive’ model of magnetic field coefficients for the timing of the last IGRF release, (ii) a predictive model of magnetic field coefficients for the timing of this IGRF release, and (iii) the predicted average magnetic field change for the following 5 years following IGRF release. IGRF is produced by a collaborative effort of the International Association of Geomagnetism and Aeronomy (IAGA) to provide a set of weighted spherical harmonic coefficients from all submitted candidates, which has led to multiple improvements in magnetic field modelling including flow inversion methodology, data assimilation techniques, and external field modelling (e.g. Brown et al. 2021; Gillet et al. 2024; Huder et al. 2020; Tangborn et al. 2021; Kloss et al. 2023). The use of extensive data sets from satellite missions like Swarm (Olsen et al. 2013), China Seismo-Electromagnetic Satellite (CSES) (Yang et al. 2021), and MSS-1 (Li et al. 2025), along with continuous data from ground-based observatories overseen by INTERMAGNET (Rasson 2007b), has enhanced the accuracy of these models. The IGRF must be updated periodically (every 5 years) using new satellite and ground-based data, as long-term forecasting remains challenging due to the complex, unpredictable nature of Earth’s core processes.

The fourteenth generation of the model, IGRF-14, covers the period from 1900.0 to 2030.0. It includes the Definitive Geomagnetic Reference Field (DGRF) for 2020.0, which replaces the preliminary main field estimate for 2020.0 provided by IGRF-13; a prediction of the MF in 2025.0; and the average SV for 2025.0 to 2030.0. The evaluation of these candidate models was coordinated by the IAGA Working Group V-MOD, which leads the dedicated IGRF task force. Readers are referred to the papers in this special issue, particularly those evaluating the candidate models, for further details on the different candidates and their comparative performance. This IGRF generation has more candidate submissions than ever before. The IGRF task force made all of the coefficients publicly available on a IGRF-14 evaluation repository on GitHub, allowing users to conduct more comparative analysis and supporting the principles of FAIR research—making research outputs Findable, Accessible, Interoperable, and Reusable (FAIR).

As previously mentioned, the dominant contribution to the magnetic field at Earth’s surface originates from flow motion in Earth’s outer core. While some IGRF candidates incorporate flow models within their construction, the IGRF itself is generally not used for this purpose. This is because other geomagnetic field models are updated more frequently and offer a higher spherical harmonic degree truncation for SV, making them more suitable for core studies (e.g. Baerenzung et al. 2022; Finlay et al. 2020). In this work, we aim to utilise the new GitHub repository to investigate the variability of core surface flow models inverted from the different IGRF candidate field models. This analysis helps to identify regions, where flow is least constrained and provide error estimates on flow based on using different geomagnetic field models that have been constructed for the same purpose. In addition, we aim to explore the impact of SV truncation degree on the resulting core flow models. The last update to the IGRF construction occurred in 2003, when the maximum spherical harmonic degree for the DGRF and IGRF main field was increased from 10 to 13, following a vote by IAGA Working Group V-8 at IAGA’s Joint Scientific Assembly with International Association of Seismology and Physics of the Earth’s Interior in 2001 (Macmillan 2003; Macmillan et al. 2003). We seek to evaluate whether improvements in satellite instrumentation and increased data coverage now justify raising the IGRF SV prediction truncation degree above the current limit of degree 8, and to assess the impact such an increase would have on the resulting core surface flow models.

This paper is structured as follows: we begin by describing the pygeodyn core flow inversion methodology and the candidate field models in Sects. 2.1 and 2.2, respectively. In Sect. 3, we present results inverted from all 13 candidate field models along with the published IGRF coefficients. Then, in Sect. 4, we examine the effect of increasing the truncation degree for the SV component of the IGRF candidate models on the resulting flow models. We conclude with a discussion of our findings in the context of IGRF construction in Sect. 5 and our summarize our main findings in Sect. 6.

## Methodology

### Core flow inversion methodology with pygeodyn

When we assume the mantle to be insulating, the MF vector $\textbf{B}$ above the core surface (of radius $c=3485$ km) is entirely defined by its radial component $B_r$, whose evolution is governed by the radial induction equation at the top of the core:

2 $$\frac{\partial B_r}{\partial t} = -\nabla_h \cdot ({\bf{u}}_h B_r) + \frac{\eta}{r} \nabla^2 \left(r B_r\right),$$

where $\eta$ is the magnetic diffusivity, $\textbf{u}_h$ is the horizontal core motion, and $\nabla _h$ is the horizontal gradient operator (e.g. Holme 2015). The high conductivity of the outer core permits the use of the ‘frozen-flux’ assumption, whereby large scale temporal changes in the magnetic field—observed over timescales of years to centuries—can be treated as a ‘tracer’ of the fluid motion at the core–mantle boundary (CMB, Backus and Le Mouël 1986; Bloxham and Jackson 1991). However, flow determination is a non-unique problem. As the inversion seeks to estimate two unknowns—the northward and eastward flow components—from a single observational constraint, the SV, additional assumptions are required to reduce this ambiguity. (Backus 1968; Roberts and Scott 1965). We refer to other studies to describe the changes in flow structure when different flow assumptions are applied (for instance Amit and Pais 2013; Beggan and Whaler 2008; Finlay et al. 2010a; Rau et al. 2000).

Thanks to advances in computational power and modelling techniques, it is now possible to utilise second-order statistics from free runs of geodynamo numerical simulations within a data assimilation framework. This allows for the use of physically consistent priors, replacing traditional assumptions about core flow (e.g. Barrois et al. 2017; Fournier et al. 2011; Sanchez et al. 2020). It is important to note that the geodynamo simulation must be run for sufficiently long duration (typically on the order of 10 kyr) to generate statistically converged outputs suitable for assimilation, and simulations must be appropriately Earth-like (Barrois et al. 2017; Sanchez et al. 2020; Rogers et al. 2025). pygeodyn is an open-source Python package that performs a sequential data assimilation algorithm (an augmented state ensemble Kalman filter, see Evensen 2003) to perform core flow inversion from geomagnetic observations using prior information derived from geodynamo simulations. This methodology has been developed over several years (Barrois et al. 2018; Gillet et al. 2024; Huder et al. 2019; Istas et al. 2023), and has been applied by multiple authors to provide insights into dynamics in the core (including wave-like motions), variations in the length of day, and the development of geomagnetic field forecasts (e.g. Gillet et al. 2019, 2022, 2024; Huder et al. 2020; Suttie et al. 2025). The augmented state ensemble Kalman filter enables the joint estimation of the state of a dynamical system, such as the core flow field, and the evolution of auxiliary variables (some unknown) that influence it. All fields are expanded in the spectral domain using spherical harmonics, and Eq. (2) in matrix form becomes:

3 $$\dot{\textbf{b}} = {\textsf{A}}(\bf{b}){\bf{u}} + {\bf{e}}, $$

where $\dot{\textbf{b}}$, $\textbf{b}$, $\textbf{u}$ and $\textbf{e}$ are vectors that store the spherical harmonic coefficients for the radial main field, the radial Secular Variation (SV), the core surface flow and the Errors of Representativeness (ER), respectively. The errors of representativeness matrix encompasses the diffusion and unresolved small-scale flow (Gillet et al. 2019) and the Gaunt–Elsasser matrix ($\textsf{A}(\textbf{b})$) relates the flow to the SV (Whaler 1986).

To enable a fair comparison between the field models, we adopt the same AR1 ‘dense’ methodology and keep the choice of prior (71% prior described in Aubert and Gillet 2021). This identical setup ensures that any differences arise from the choice of candidate field model, rather than from the choice of prior or methodology (Rogers et al. 2025). We perform the flow forecast with an ensemble of 200 models in 1-month steps and conduct the flow analysis in 1-year increments over the period 2020.0$-$2030.0. The maximum truncation degree of flow ($L_U$) and MF ($L_B$) are chosen to be 15 and 13, respectively. To mitigate noise arising from empirical covariance estimation, the graphical Lasso parameter (described in Istas et al. 2023) is set as 0.15. The choice of $L_B$ follows directly the truncation degree of the IGRF. In contrast, the choice of $L_U$ is somewhat arbitrary, chosen as a trade-off between a risk of aliasing and the wish to limit the dimension of the unknown vector (flow coefficients of degrees much larger than $L_B$ being unresolved, see Baerenzung et al. 2016; Barrois et al. 2017). The matrix for the errors of representativeness is truncated to the same spherical harmonic degree as the SV, discussed in the next section.

### Field models

**Table 1:** Table summarizing the different construction and data used by each candidate models used in this study When precise data set details are not specified in the candidate technical notes, it may indicate that both Vector Field Magnetometer (VFM) and Absolute Scalar Magnetometer (ASM) data were used, or that the information was not provided. For full model construction details, see the submitted manuscripts in this special issue and the candidate technical notes

Abbreviation	Leading Institution, Country	Satellite data	Observatory data	Other data	Model construction
Huber	IAGA, International	None	None	Candidate Models	Median of DGRF candidate models and Huber-weighted mean in space of IGRF and SV candidate models
BGS	British Geological Society, UK	Swarm, MSS-1	Hourly mean vector data and quasi-definitive and near-definitve data	None	Spline-based time-dependent core field model, static crustal field model with corrections for observatories, spline-based large-scale slowly varying external field, spline-based large-scale rapidly varying external and induced field model, external and induced periodic variations
CSES	National Institute of Natural Hazards, China	CSES	None	None	Spline-based time-dependent core field model, static crustal field model, static and time-dependent external field models, Euler angles
DTU	Danish Technical University, Denmark	Ørsted, CHAMP, SAC-C, CryoSat-2, Swarm, CSES, MSS-1	Annual differences of revised monthly means of vector data	Temporal covarainces from geodynamo simulations	Spline-based time-dependent core field model with acceleration regularised with geodynamo a priori information, static crustal field model, static and time-dependent external and induced field model (ionospheric and magnetospheric), Euler angles
GFZ	GFZ Potsdam, Germany	Swarm, CHAMP L3, CryoSat-2, GOCE	Hourly mean vector data	None	Iterative re-Huber-weighted least squares scheme with temporal modelling using splines, static crustal magnetic field, spline-based slowly and rapidly varying external and induced field models, Euler angles
IPGP	Institut de Physique du Globe de Paris, France	Swarm-Alpha vector data	Hourly mean vector data	Gaussian distributed mean and covariance matrix from geodynamo	Linear Kalman filter for constant main field, constant secular variation, static lithospheric field, static external field, $D_{st}$ dependent external field and induced field, internal induced field, observatory offsets
ISTERRE	Institut des Sciences de la Terre, France	Geomagnetic Virtual Observatories from CHAMP, Ørsted, GRACE, CryoSat-2 and Swarm	3 component 4-monthly SV and MF means	a-priori information from geodynamo simulations	Order 3 auto-regressive process for internal field model and correlation function for external field, temporal modelling using splines
ITES	Institut Terre et Environnement de Strasbourg, France	None	Annual differences derived from observatory monthly means	None	Spline-based time-dependent field model with multivariate singular-spectrum-analysis
MISTA	Macau Institute of Space Technology and Application, Macau	MSS-1 and Swarm	Annual differences of revised monthly means of observatory data	CM6 ionospheric field model and CHAOS-7 geomagnetic field model	Spline-based time-dependent internal field, static crustal field, and large scale magnetospheric field
NOAA	National Oceanic and Atmospheric Administration, USA	Swarm Alpha and Bravo vector data	None	Lithospheric MF7 model and magnetospheric model from CHAOS–7.18	Iteratively re-Huber-weighted least squares with temporal modelling using splines
TUB	Technische Universität Berlin, Germany	Swarm Alpha and Bravo	Definitive or quasi definitive minute-resolution data for magnetic field and secular variation	Gaussian white noise using covariances of the core field, lithospheric field and SV	Sequential Kalman filter approach for core field, lithospheric field, induced/residual ionospheric field, remote magnetospheric field, near Earth magnetospheric field, fluctuating magnetospheric field, and a source associated with field-aligned currents
UCM	Universidad Complutense de Madrid, Spain	Swarm scalar and vector data	Hourly mean vector data and definitive and quasi-definitive minute data	None	Iterative Newton type-algorithm for linearly time dependent core field model, static crustal magnetic field model, time dependent near and remote external field models
USTHB	University of Sciences and Technology Houari Boumediène, Algeria	Swarm Alpha	None	None	Time-dependent and static internal field components, and time dependent induced and external fields for set time periods. Linear extrapolation of SV prediction
WHU	Wuhan University, China	Swarm Alpha and Bravo, GRACE-FO and CryoSat-2	None	External and lithospheric field model from CHAOS–7.18	Non-linear least-square fitting algorithm for internal field modelling with temporal modelling using splines

We consider all 13 candidate groups that submitted the three constituent models to the IGRF repository: a DGRF, IGRF and SV. In addition, we include the published IGRF model constructed from the median of the candidate DGRF models and the Huber-weighted in mean space for IGRF and SV candidates. Table 1 provides a brief summary of the 14 models we compare. For further details on each model construction and data selection, we refer the reader to the relevant papers in this special issue.

**Fig. 1:**
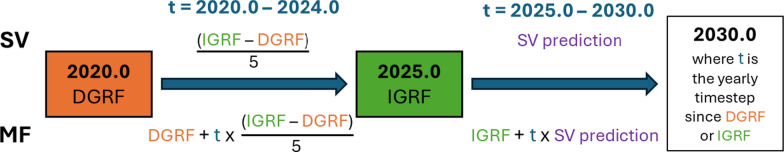
Schematic showing how the yearly values for the Secular Variation (SV) and Main Field (MF) were calculated from the DGRF, IGRF and SV prediction for each candidate model. The IGRF and DGRF are given as Schmidt semi-normalized Gauss coefficients with SV and MF values between 2020.0 and 2025.0 truncated at degree 13. Between 2025.0 and 2030.0, the predicted SV is truncated at degree 8 and the MF for degrees 9–13 is held constant

Each considered candidate model provides Gauss coefficients describing the MF model truncated at degree 13 ($L_B=13$) in 2020.0 and 2025.0 as well as a prediction of the average SV for 2025.0 to 2030.0 truncated at degree 8 ($L_{SV}=8$). To produce yearly data points we follow the procedure illustrated in Fig. 1, assuming a steady SV between the DGRF and IGRF epochs (2020.0 and 2025.0) and again from 2025.0 to 2030.0, consistent with the end of the SV prediction period (2030.0). As DGRF and IGRF $L_B=13$, the steady SV we model for 2020.0$-$2025.0 has a maximum truncation degree of 13, but a maximum truncation degree of 8 for the prediction between 2025.0$-$2030.0. As a steady SV enters into our flow modelling, the resulting flow model also acts similar to a steady flow. The pygeodyn code requires that $L_{SV} \ge L_B$. To satisfy this constraint when $L_{SV} < L_B$, we set the SV coefficients to zero for degrees $l = 9$ to 13 and assign them extremely large uncertainties (order of magnitude larger than the largest coefficient value), effectively removing their influence on the solution.

In Sect. 4 we examine the effect of increasing $L_{SV}$ for the SV prediction over the period 2025.0$-$2030.0. To do this, we compile the SV coefficients for the DTU candidate (also known as CHAOS–8.1) and TUB candidate (also known as Kalmag) at $L_{SV}=13$, using data from their respective online repositories. This process is simplified by the fact that DTU defines the SV prediction as instantaneous value of SV at 2024.0 and TUB defines it as the instantaneous value of SV at 2025.0. The coefficient values of the MF between 2025.0 and 2030.0 are the same for $l=1-8$ for $L_{SV}=13$ or $L_{SV}=8$.

## Comparing flow models from all candidate models

**Fig. 2:**
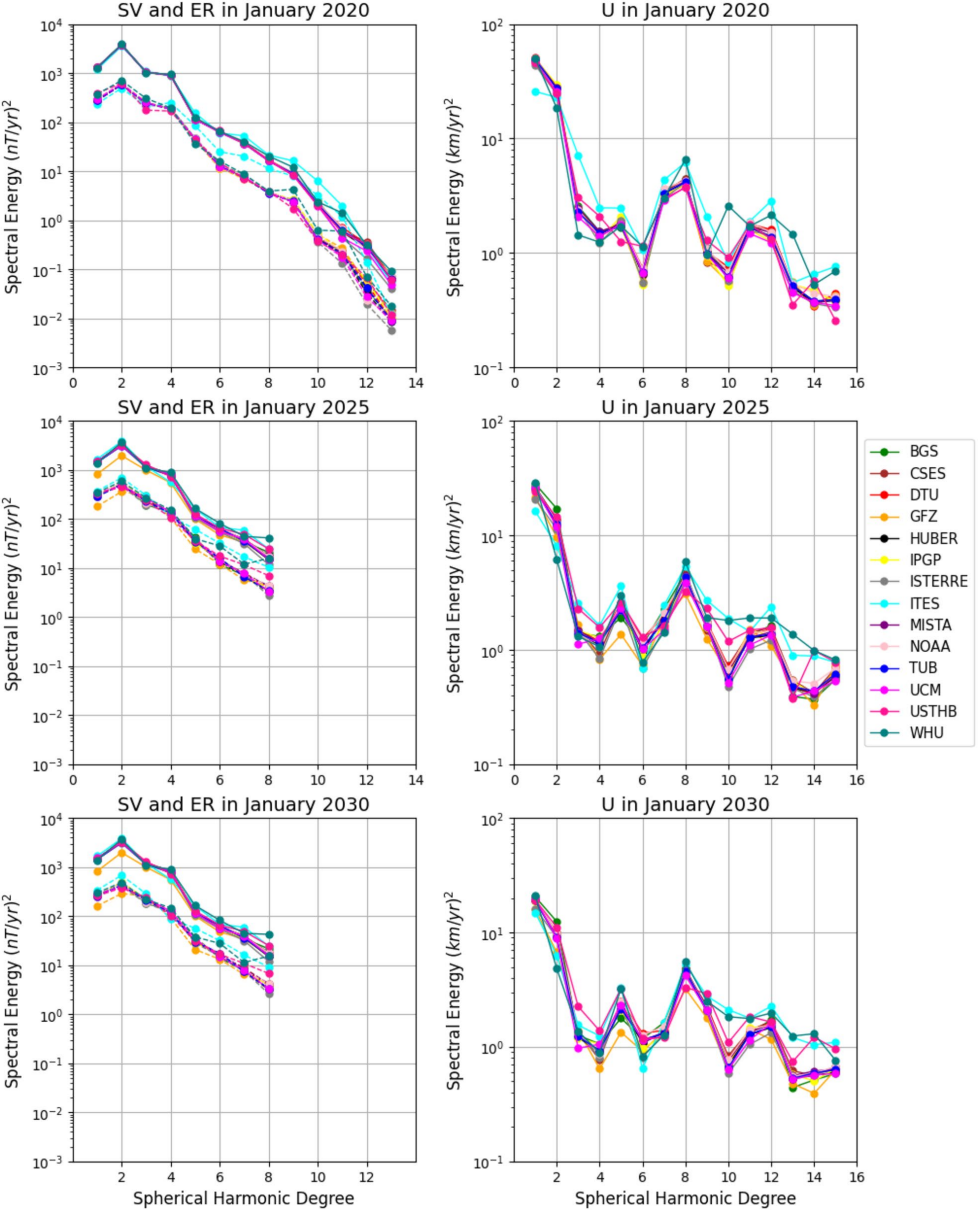
Spectra of the Secular Variation (SV, solid) and Errors of Representativeness (ER, dashed), left, and the inverted flow model (U), right, for each candidate model for 2020.0 (DGRF epoch, top), 2025.0 (IGRF epoch, middle) and 2030.0 (end of SV prediction epoch, bottom)

First, we compare the flow model outputs from the pygeodyn core flow inversion code using the various IGRF-14 candidate models. Figure 2 presents the spatial spectra at Earth’s surface of the SV and errors of representativeness (as defined by Lowes (1974), left) as well as flow spectra (as defined by Le Mouël et al. (1985), right) for all candidate models in 2020.0 (top), 2025.0 (middle) and 2030.0 (bottom). These three dates correspond to the DGRF epoch, the IGRF epoch and the end of the SV prediction period, respectively.

In 2020, the SV spectra are nearly identical for all degrees up to degree 10, with deviations becoming more apparent at higher degrees. These differences are likely to be due to the choice of data selection and candidate construction for DGRF and IGRF models, affecting the small scale features in the MF models. Notably, the spectra of the errors of representativeness (which account for subgrid induction and diffusion) exhibit greater variability than the SV spectra, even at low degrees. Some aliasing may have occurred when co-estimating the errors of representativeness with the flow using the augmented state ensemble Kalman filter. To mitigate this and in the light of the low truncation of $L_{SV}$ we opted for a lower $L_U$ than has been used in some previous studies (e.g. Gillet et al. 2022; Rogers et al. 2025).

The ITES model shows the largest variations in SV spectra above degree 5, and we also observe that the WHU candidate shows increased energy in degrees 8–13. ITES is solely based on ground observatory data, which highlights the importance of satellite observations on constraining higher degree SV, as it is the model with the largest deviation from the candidate mean in the IGRF. The WHU model is also an outlier model in both the IGRF and DGRF, with larger deviations in the magnetic field concentrated near the geomagnetic poles and in clear zonal bands. These features may be due to the choice of satellite data, as WHU is the only candidate incorporating data from GRACE Follow-On, though this requires further investigation. Surprisingly, the CSES, ISTERRE, and USTHB candidates show no deviations in the flow spectra below degree 11, despite appearing to be far from the candidate median in DGRF and IGRF. This may be because the models were constructed in the same way for both 2020.0 and 2025.0, leading to a constant model offset from the Huber weighted mean and resulting in a similar SV to the other models.

At 2025.0 and 2030.0, the SV spectra are identical and truncated at $L_{SV}=8$ as both epochs use the same $L_{SV}=8$ SV prediction. The SV spectra show greater spread among the different candidate models across all degrees, which we attribute to the different forecasting methods and the lack of data constraints. GFZ shows the lowest SV spectral energy, particularly noticeable at degrees $l=1-2$. The variation between candidate models increases with spherical harmonic degree and is largest at degree 8. The ITES model is one of the most dissimilar, the large variation seen in 2020.0 is no longer observed. Although the SV is identical between 2025.0 and 2030.0, the spectra for the errors of representativeness are not. The errors of representativeness increased slightly between 2025.0 and 2030.0, potentially reflecting the compounding of the aliasing due to the large error values assigned to SV degrees 9–13 over time.

The flow spectra of all candidate models at epoch 2020.0 exhibit a similar overall trend, though they show greater variability compared to the SV spectra. As in the SV case, the ITES model stands out as a clear outlier, displaying low flow spectral energy at $l=1$, but elevated kinetic spectral energy at degrees higher than 2. The WHU model shows low flow spectral energy in degrees 2–4 and high spectral energy at degrees 10–15. The USTHB is an outlier for certain degrees (3–6, 10, 13–15), though to a lesser extent than ITES and WHU models. The flow spectra in 2025.0 and 2030.0 differ slightly from each other; however, they remain very similar due to assumption of steady SV between 2025.0 and 2030.0. These later epochs show less variation than 2020.0, with the GFZ model displaying the lowest kinetic spectral energy, and the WHU and ITES models continuing to show significant disparities relative to the other models. The flow energy at low degrees ($\le 8$) decreases progressively from 2020.0 to 2025.0 to 2030.0, as a result of the lower $L_{SV}$ used in later periods.

**Fig. 3:**
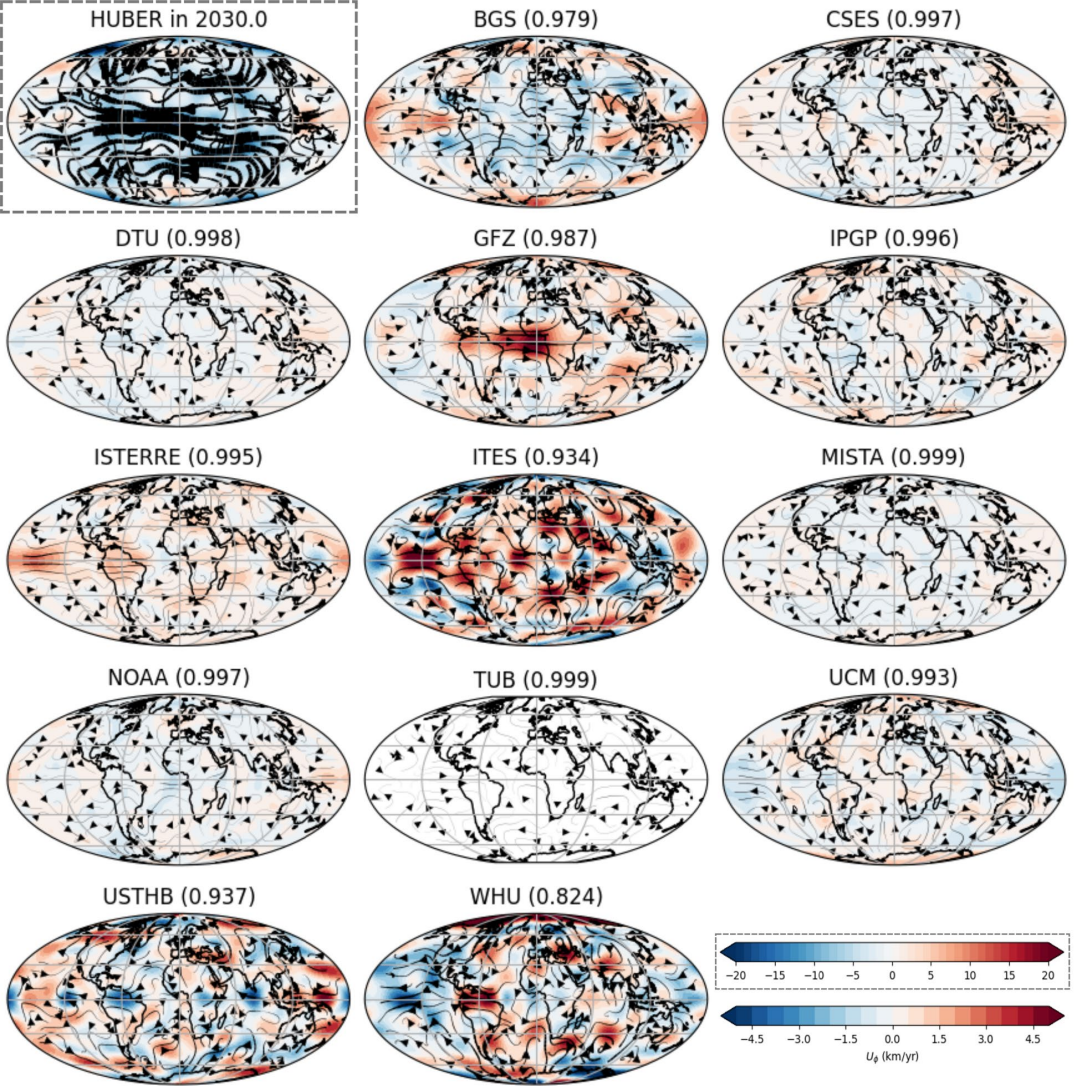
Predicted flow for the 2030.0 timestep for the published Huber-weighted mean model in space IGRF model (shown within grey dashed box) along with the difference between each candidate model and the published IGRF model. The number in the brackets after each candidate name indicates the correlation coefficient between the $U_\phi$ of the flow model inverted from the Huber-weighted mean model in space and $U_\phi$ of that of the corresponding candidate model. The colorbar for all models are consistent and shown for in the bottom right corner, except for the weighted mean model in space which is shown in its own grey dashed box

In Fig. 3, we present the flow maps of the Huber-weighted mean model in space at each epoch, along with the difference of each candidate with respect to the Huber weighted model in the 2030.0 epoch (equivalent maps for 2020.0 and 2025.0 epochs are provided in Appendix 1). Most candidate models are similar to the Huber weighted model at all epochs, with many showing flow speed differences less than 5% of the maximum flow speed of the Huber weighted model. In 2030.0, all models show high correlation coefficient values ($\ge 0.82$), though the models become less correlated at the IGRF ($\ge 0.74$) and DGRF ($\ge 0.73$) epochs. Although this may seem counter-intuitive, the lower $L_{SV}$ for 2030.0 reduces the spectral energy for the flow and suppresses small-scale differences, leading to more similar flow structures across the candidate models.

In 2030.0, the TUB candidate model is the most similar to the IGRF-14 model inversion, with a root mean square error (RMSE) of 0.31 km/year and a correlation coefficient of 0.999. The WHU exhibits the largest flow deviation, reaching 14.2 km/year, while the RMSE are also relatively high for ITES (2.4 km/year), WHU (3.1 km/year), and USTHB (2.1 km/year). The ITES and USTHB primarily show small scale flow variations distributed across the entire surface, whereas the WHU flow model displays its most intense deviations in the polar regions and near the equator in the western hemisphere. The BGS, GFZ and ISTERRE models show variations at the equator: BGS and ISTERRE exhibit stronger eastward flow in the Pacific region, while GFZ shows enhanced eastward flow in the Atlantic. These candidate models have intense SV differences at the equator compared to the Huber weighted mean model, which is reflected in their flow outputs.

**Fig. 4:**
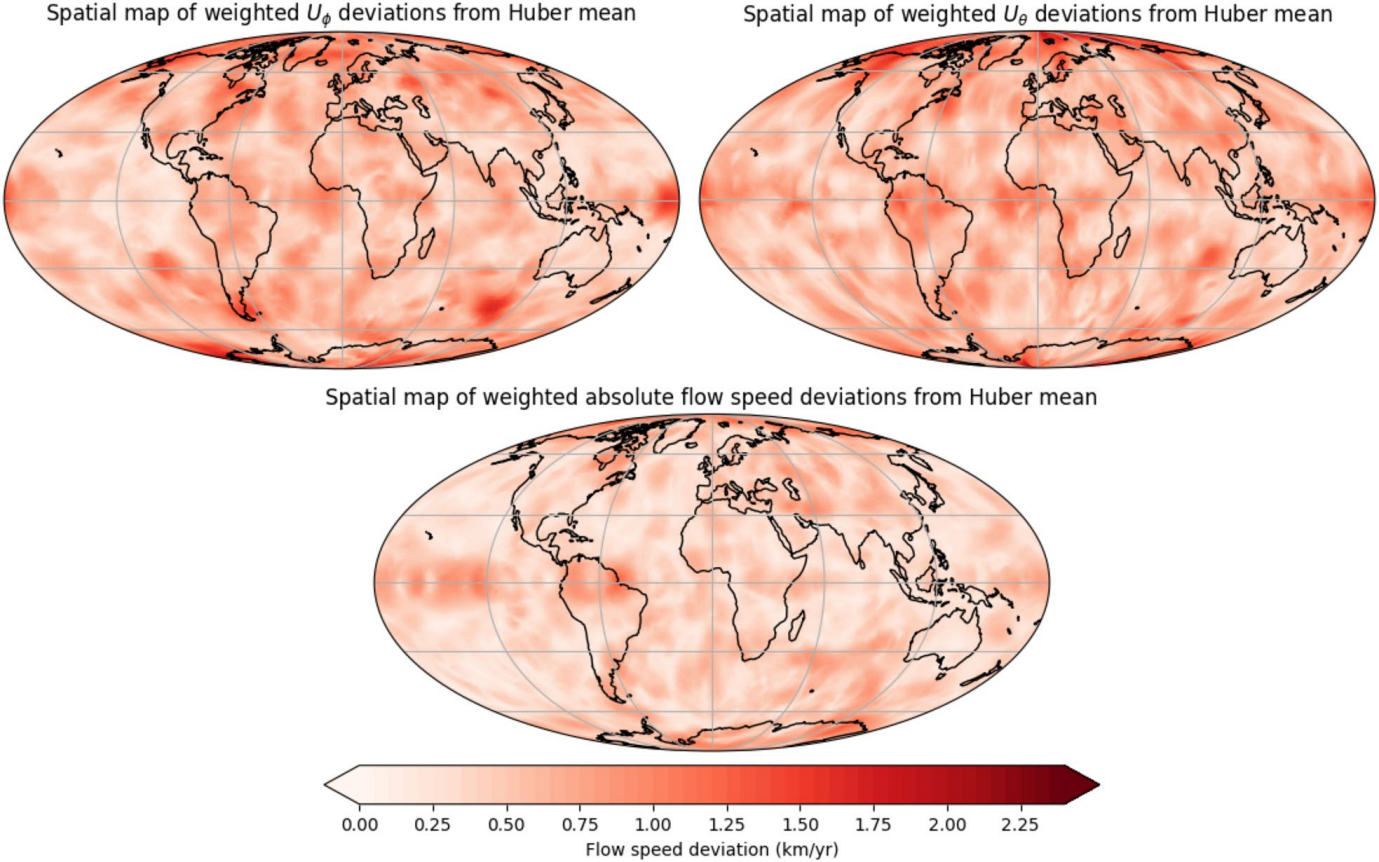
Spatial Huber weighted mean of the deviation of flow from the flow inversion (shown in Fig. 3) in the west–east direction ($U_\phi$, top left), north–south direction ($U_\theta$, top right) and absolute flow speed (bottom)

Figure 4 shows the Huber weighted deviation from the mean for the flow in the west–east ($U_\phi$), north–south ($U_\theta$) and absolute flow speed components. The flow speed uncertainty is greater for the individual scalar directions than for the absolute flow speed, and uncertainty is higher for $U_\phi$ than $U_\theta$. At the equator, deviations are more pronounced in the Pacific hemisphere than in the Atlantic, particularly for the absolute flow speed, but this does not hold true at higher latitudes. The flow direction ($U_\phi$ than $U_\theta$) is poorly constrained near the northern pole, but all weighted flow deviation values are small, indicating that the overall flow structure is consistent across most models. In the polar regions, the higher uncertainty is instead due to the intrinsic geometric limitations of resolving tangential flow from the available field observations and external field contaminations in the data. Applying the Huber weighted deviation down-weight the models that are most dissimilar, meaning that the ITES, WHU, and USTHB deviations in Fig. 3 become reduced.

Despite the high correlation between all candidate models, the maximum Huber weighted deviation from the maximum velocity of the Huber weighted mean is relatively high for individual directions, 25.1% for $U_{\phi }$, 19.7% for $U_{\theta }$, but lower for the absolute flow speed, 5.0%. The Huber weighted deviation is smaller than the mean standard deviation of the ensemble members of the Huber model (51.1% for $U_{\phi }$, 35.9% for $U_{\theta }$, 23.8% for absolute flow speed), meaning that the distribution of each model is probably overlapping. The time-averaged $71\%$ path dynamo prior is heterogenously forced but the patterns of greatest uncertainty does not match the time-average prior. Suttie et al. (2025) and Rogers et al. (2025) have shown that the choice of prior can influence flow structure; therefore, future work could investigate whether these flow uncertainties remain consistent when varying the geodynamo prior in the pygeodyn methodology.

**Fig. 5:**
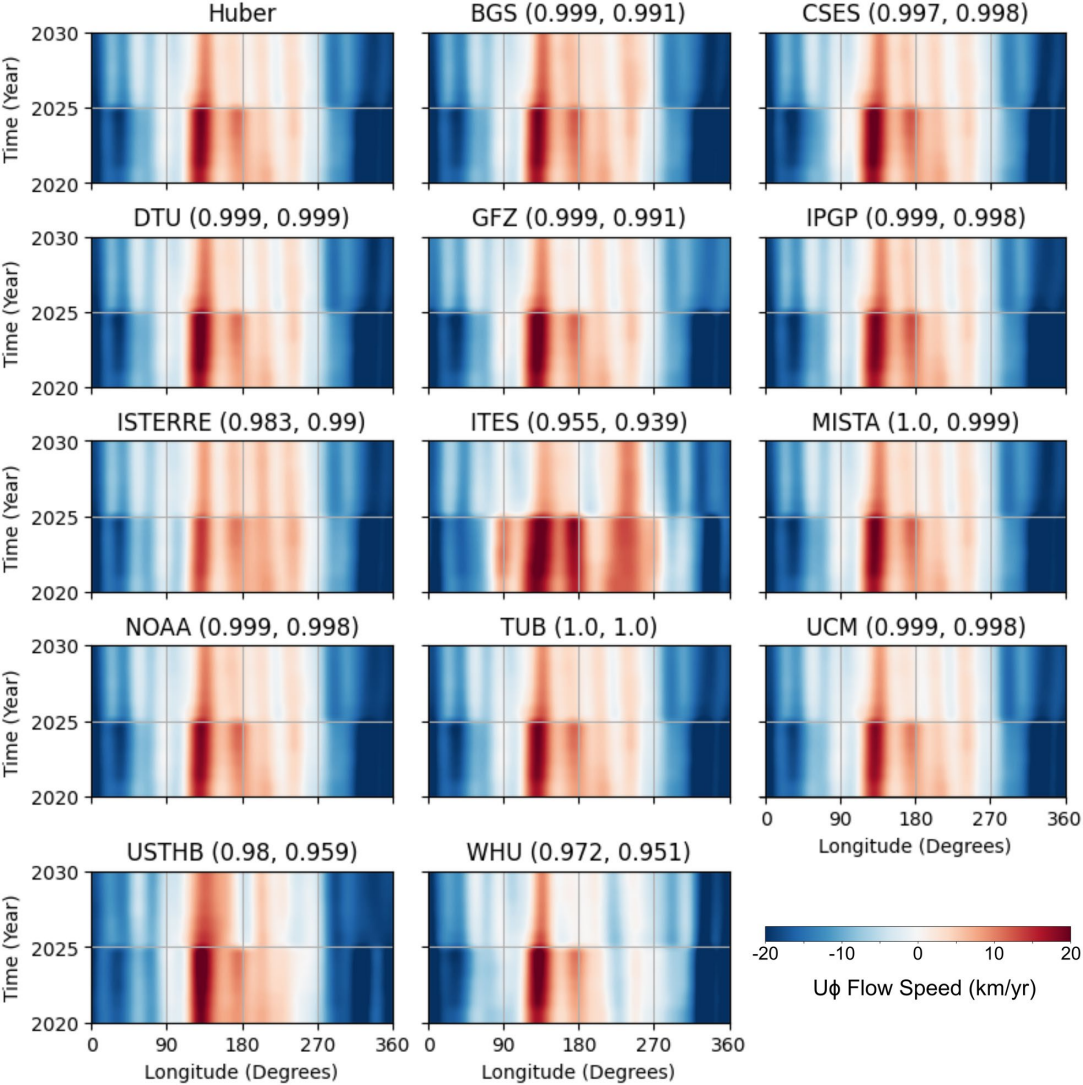
Time-longitude plots of $U_\phi$ at the equator over the 10-year period covered in the study. The numbers after the candidate model name indicate the correlation coefficient in 2023 and 2028, for the equatorial $U_\phi$ from the candidate flow model to the published Huber-weighted mean model in space

In Fig. 5, we plot the east–west ($U_\phi$) flow speed at the equator over time, using a time-longitude diagram. As mentioned in Sect. 2.2, we assume a steady SV, because we only have two timepoints for the Gauss field coefficients (DGRF and IGRF) along with a prediction of the average SV. This resulting steady flow is evident from the consistent vertical stripes between 2020.0 and 2025.0, and between 2025.0 and 2030.0 with a sharp transition in 2025.0. Part of this transition reflects the weakening of the flow speed after 2025.0, due to the decrease in $L_{SV}$ from 13 to 8. All time-longitude plots share a similar pattern with the fastest eastward flow at $135^\circ$ and the fastest westward flow between 315° and 10°. The ITES candidate flow stands out for 2020.0$-$2025.0 exhibiting intense eastward flow near $90^\circ$ and $245^\circ$ which is much weaker in the other models.

When calculating the correlation coefficient for the equatorial $U_\phi$ in 2023 and 2028 for all candidate models, two interesting characteristics emerge. First, the correlation for the equatorial $U_\phi$ is higher than the correlation obtained from comparing global flow maps, indicating that the greatest variability among flow models occurs in the polar regions—an observation also noted by the IGRF task force’s evaluations. Second, some flow models show greater variation at the equator in 2028.0 than 2023.0. While this might seem to contradict the findings from the global flow maps, the correlation remains extremely high at both times, and Fig. 4 shows more flow variability at the equator compared to mid-latitudes. The lower spherical harmonic degree truncation in 2028.0 leads to larger spatial scale flow in the time-longitude plots (e.g. USTHB).

## Choice of spherical harmonic degree truncation on core flow inversion

**Fig. 6:**
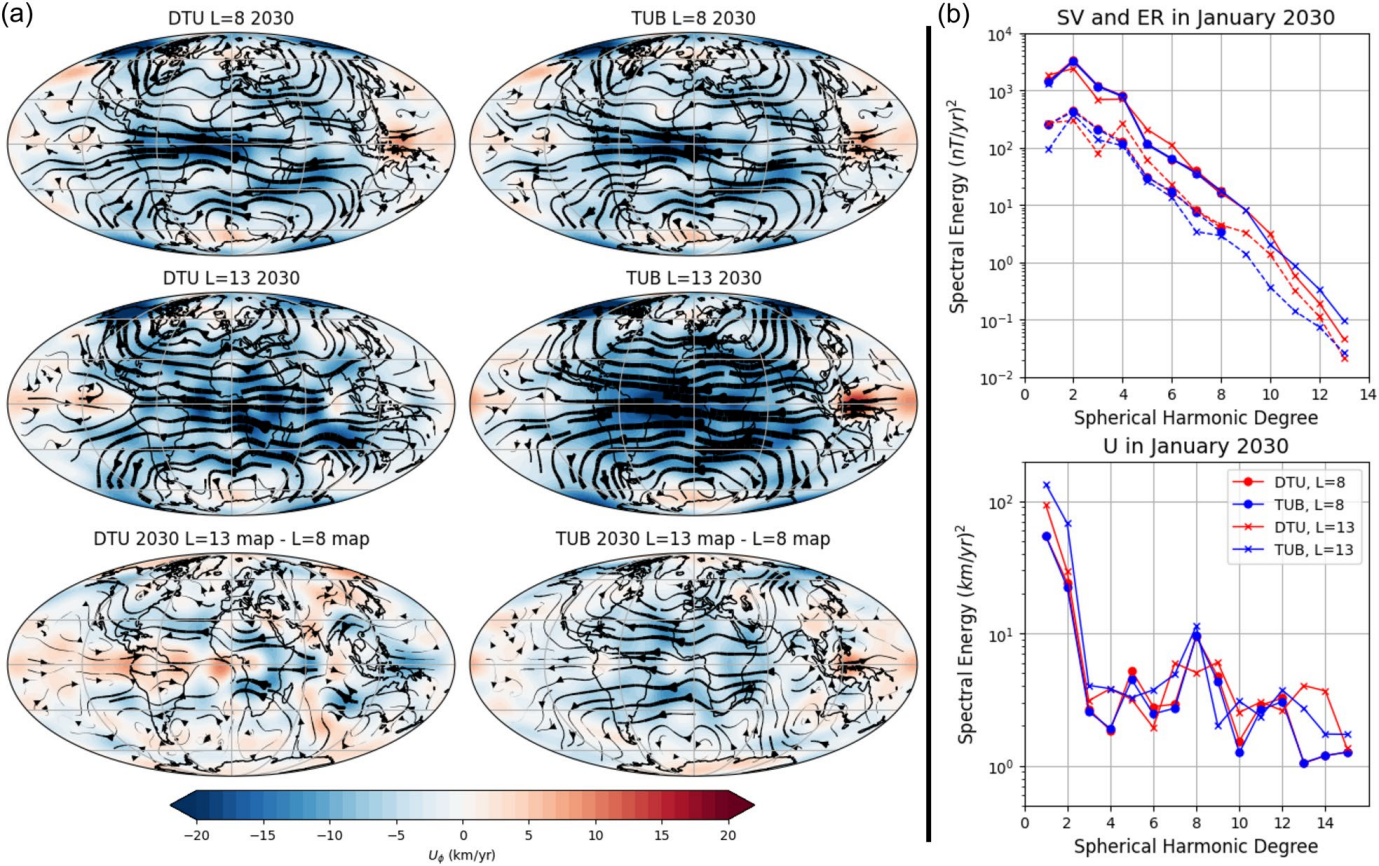
Difference in the flow maps (**a**) and the SV and flow spectra (**b**) for the DTU and TUB candidates truncated at spherical harmonic degree 8 and 13 for the 2030.0 epoch. The colorbar for (**a**) is constant for all subplots. In panel (**b**, bottom) we also show the ITES candidate truncated at $L_{SV}=8$ for comparison with the spectra shown in Fig. 2

We have already noted that the lower $L_{SV}$ for 2025.0$-$2030.0 results in reduced spectral energy for the flow spectra and slower maximum flow speeds in the flow maps. The DTU and TUB candidates (also known as CHAOS–8.1 and Kalmag, respectively) publish coefficients on their respective websites and, allowing us to compare the effects of truncating the SV prediction coefficients to degree 13 against the published IGRF coefficients (truncated at degree 8). Figure 6 shows the resulting flow models and spectra for these two candidates at $L_{SV}=8$ and $L_{SV}=13$ in 2030.0. We do not repeat the flow inversions in 2020.0, as $L_{SV}$ is already 13 at that epoch, nor for 2025.0, due to the similarities in spectra and flow maps with those discussed in Sect. 3.

First, Fig. 6(a) shows that increasing $L_{SV}$ to 13 increases the flow speed of the model. The difference in flow between $L_{SV}=13$ and $L_{SV}=8$ is larger than that between the different candidates in Sect. 3. Specifically, the maximum absolute flow speed increases by 4.0 and 30.8% for DTU and TUB, respectively. Increasing the DTU $L_{SV}$ primarily enhances eastward flow in the equator, whereas increasing the TUB $L_{SV}$ strengthens westward flow at the equator, resulting in a more pronounced increase in the maximum absolute flow speed. Despite these differences, the correlation coefficient between the $L_{SV}=8$ and $L_{SV}=13$ maps remains high (0.783 and 0.871 for DTU and TUB, respectively).

Figure 6(b, top) indicates that the SV spectra and the corresponding errors of representativeness differ in behaviour for the DTU and TUB candidates when truncated at $L_{SV}=8$ and $L_{SV}=13$. The TUB SV model for $L_{SV}=13$ perfectly matches the values of TUB $L_{SV}=8$ for $l=1-8$, extended to $L=13$. Despite this apparent agreement, the errors of representativeness for TUB $L_{SV}=13$ differ from the TUB $L_{SV}=8$, particularly at $l=1$ and $l=7$. Meanwhile, the DTU SV model and errors of representativeness for $L_{SV}=13$ vary from the $L_{SV}=8$ model. It is also worth noting that in DTU model the errors of representativeness for $l=10-13$ comprise a large proportion of the SV, which is not expected to be realistic in the core (Backus and Le Mouël 1986; Gillet et al. 2019). These observations suggest that aliasing may have been introduced, likely due to the proximity of $L_U$ to $L_B$ (Barrois et al. 2017). As previously discussed, the choice of $L_U$ is arbitrary, except from the requirement that $L_U > L_B$. Low-degree $L_{SV}$ coefficients can differ during the inversion due to coupling with higher degree terms with both the SV and ER terms. The degrees at which the SV spectral energy deviate correspond to the degrees at which there is a change in the spectral energy of ER, leading to the observed differences. Determining an optimal value for $L_U$ becomes particularly challenging when $L_{SV}$ is low, especially when comparing the candidate models ($L_{SV}$=8) and attempting to keep the values consistent between our two studies.

Figure 6(b, bottom) shows that, despite the differences in the flow map presented in panel (a), the overall shape of the flow spectra remains similar, with two key differences. First, the $L_{SV}=13$ models have a noticeably higher energy in the lowest spherical harmonic degrees ($l=1-4$, particularly $l=1$) which are the dominant degrees in the spectrum, resulting in the faster flow speeds observed in Fig. 6(a). Second, the $L_{SV}=13$ models have more kinetic energy in the smallest spatial degrees ($l=13-15$). It is important to note that the large and small scale flow and magnetic field features interact to produce SV at all spatial scales (Pais and Jault 2008). Increasing the SV prediction increases the resulting MF in 2030.0, thereby affecting the flow inversion at all spatial scales. However, increases in small scale SV are more likely effective to increase the energy of small scale flows.

Finally, we also include the ITES $L_{SV}=8$ candidate in the flow spectra of Fig. 6(b) as it was previously identified as an outlier in Sect. 3. The ITES candidate continues to stand out as it has low spectral energy in degrees 1 and 2. In fact, its differences from the TUB $L_{SV}=8$ model are often greater than the two TUB models themselves. This is only true for some degrees of DTU ($l=10-12$). These observations suggest that data selection and SV model construction play roles as significant as the choice of the maximum truncation degree in the resulting flow model. The correlation coefficient of $L_{SV}=8$ ITES to $L_{SV}=8$ Huber-weighted mean model in space (0.934) is higher than the $L_{SV}=13$ models (DTU = 0.786 and TUB = 0.866). Notably, the TUB $L_{SV}=13$ model shows a greater correlation to the Huber weighted model than the WHU $L_{SV}=8$ candidate in 2030.0 (0.824).

## Discussion

A key milestone in the IGRF model’s history occurred with the release of IGRF-9 in the year 2000 (Macmillan 2003; Macmillan et al. 2003), when the maximum degree of the spherical harmonic expansion for the MF was increased from 10 to 13. This enhancement significantly improved the model’s ability to capture smaller scale features of the Earth’s magnetic field, reducing the minimum resolvable wavelength at the Earth’s surface from roughly 4000 km to approximately 3000 km (Mandea and Macmillan 2000; Lowes 2000).

The increase in resolution for both the IGRF and DGRF was enabled by several developments. First, the improved data quality, especially due to the Ørsted satellite (launched in 1999), which provided high-precision, global measurements of the geomagnetic field (Olsen et al. 2000). As noted by Mandea and Macmillan (2000), the integration of satellite observations into the IGRF significantly enhanced its accuracy and spatial detail, marking a shift in both methodology and expectations for geomagnetic reference models. Second, expanded coverage from ground observatories, offering a more reliable global data set in the frame of INTERMAGNET programme (Rasson 2007a; Kerridge 2001). Finally, significant advances in computing power allowed more complex inversion techniques and the robust estimation of higher degree spherical harmonic coefficients (Lesur et al. 2008). The adoption of degree 13 has since remained a standard in all subsequent IGRF releases, reflecting a well-judged balance between spatial resolution and model reliability.

While the IGRF models the MF up to spherical harmonic degree 13, its representation of SV remains limited to degree 8. This disparity reflects a historically conservative approach to modeling temporal changes, largely justified by earlier limitations in data resolution and global coverage. The last formal vote on increasing the $L_{SV}$ was in 2009 (Finlay et al. 2010c). Silva et al. (2010) found that increasing $L_{SV}$ to at least degree 10, or even 12, could produce predictive SV models with comparable skill to the existing degree 8 forecasts. Moreover, they found that degrees beyond 8 contribute relatively little to total spectral power, and their omission introduces less error than the uncertainties already present in the lower degrees, consistent with the red spectral character of Earth’s SV at the surface. However, Silva et al. (2010) placed strong caveats on this finding, including: (i) continued availability of high-quality data provided by on-going satellite missions coverage (in this case CHAMP); and (ii) the model must be restricted using past SV over a short enough period of time. These conditions suggest that models built on solely observatory data (e.g. IEST candidate model) would be down-weighted in future IGRF releases relative to satellite and observatory-based models. While observatory-only models may not resolve the full spatial complexity achievable with satellite data, observatory measurements continue to play a crucial role in model validation, long-term continuity, magnetic indices, and capturing rapid field variations at specific locations (Olsen et al. 2010; Matzka et al. 2021; Olsen and Mandea 2007).

Dedicated geomagnetic satellite missions over the last two decades and ongoing advancements strongly support the need to revisit the constraint of the truncation degree for the predicted SV. In particular, the ESA Swarm mission has enabled continuous, high-resolution monitoring of MF and SV at finer spatial and temporal scales than previously possible (Friis-Christensen et al. 2006; Olsen et al. 2017; Sabaka et al. 2018; Kloss and Finlay 2019). Beyond Swarm, a new generation of missions is further expanding our capabilities. The CSES satellite and its successors provide complementary data in Sun-synchronous orbits, improving high-latitude and diurnal coverage (Yang et al. 2021). The Macau Science Satellite-1 (MSS-1), launched in 2023, is equipped with high-sensitivity magnetometers and has further improved data quality over the equatorial and low-latitude regions (Li et al. 2025). Looking forward, ESA’s NanoMagSat mission is expected to continue high-resolution magnetic field observations using low-cost nanosatellite technology (Deconinck et al. 2025; Hulot et al. 2018). With potential constellation configurations, NanoMagSat could further improve temporal sampling and spatial coverage. In addition, platform magnetometers aboard non-geomagnetic dedicated scientific satellites such as CryoSat and GRACE-FO are now being utilised to improve temporal coverage of the geomagnetic data (Stolle et al. 2021; Olsen et al. 2020; Olsen 2025).

Despite these multiple advances that suggest the truncation degree of SV in the IGRF model should be increased, there are some important considerations to be addressed. Firstly, the IGRF is the product of an international collaboration, and any modification to its construction must be approved by the IAGA V-MOD working group. Furthermore, all IGRF models contain a ‘health warning’ and previous magnetic field forecasts have been re-released due to failing to meet success criteria due to the unpredictable and chaotic nature of core dynamics (Lowes and IAGA Working Group V-MOD 2022; Chulliat et al. 2019). IGRF’s predictions are not designed for core flow inversions and must remain appropriate for their intended use. There is also ongoing work to estimate uncertainties of the IGRF and its candidate models but with mixed results. Some candidates use hindcasting, standard deviation or previous IGRF performance to calculate the uncertainty in IGRF coefficients (e.g. Beggan 2022; Brown et al. 2021; Lesur et al. 2010; Lowes 1990; Lowes and Olsen 2004; Lowes 2000). While the IGRF-14 call encouraged the inclusion of uncertainty estimates, it was not a strict requirement. It may be timely to revisit the SV degree investigations of Silva et al. (2010) using new data constraints and compare the findings with those derived from improved uncertainty estimates. Finally, any proposed changes to the IGRF construction must also demonstrate long-term sustainability, with continued international commitment to managing geomagnetic observatories and dedicated geomagnetic satellites.

On balance, we argue that given the growing wealth of high-quality satellite data, there is a strong rationale for raising the maximum spherical harmonic degree of the IGRF SV model from 8 to 13, aligning it with the MF model. In addition, advances in computational techniques and methodological improvements (e.g. data assimilation) enable robust estimates of SV at higher degrees by mitigating the risk of overfitting, leading to physically meaningful results. Increasing $L_{SV}$ would enhance IGRF ability to better capture localized, time-varying geomagnetic features, including regional features and rapid SV events such as geomagnetic jerks (Brown et al. 2013; Gillet et al. 2022; Whaler et al. 2022), and improve forecasts of the geomagnetic field, which are critical for satellite navigation, space weather prediction, and broader Earth system science.

## Conclusions

This study has investigated core surface flow variability when inverted from the IGRF-14 candidate models with the pygeodyn methodology. While differences exist among the resulting flow models, their inter-model correlations remain high ($\ge 0.73$). This is particularly true when considering $U_\phi$ at the equator ($\ge 0.95$). Areas where the flow models deviate from the model inverted from the published IGRF model can be attributed to the data and model choices in the construction of the candidates. These deviations highlight regions where candidate submissions diverge, notably in the polar regions and the equatorial Pacific, as shown by the Huber-weighted mean in space of the deviations.

Also, we have investigated the effect of the SV truncation degree on the core flow inversion. All candidate models show reduced flow speed and spectral kinetic energy at the transition at 2025.0, where $L_{SV}$ is reduced from $L_{SV}=13$ to $L_{SV}=8$. To investigate this further, we collated the DTU and TUB candidate coefficients at $L_{SV}=13$. Figure 6 shows that the $L_{SV}=13$ models display greater absolute flow speed and more variation from the published Huber-weighted IGRF model than the $L_{SV}=8$ candidate flow models. Nonetheless, the correlation for the $L_{SV}=13$ models show greater correlation (DTU = 0.786 and TUB = 0.866) than some $L_{SV}=8$ candidates to the Huber weighted mean at the IGRF and DGRF epochs ($\ge 0.73$). Notably, both the DTU and TUB models are close to the candidate mean for all three parts of the IGRF model construction. However, increasing the $L_{SV}$ may mean that correlation coefficients could decrease with outlier models, particularly those constructed using only observatory data. In conclusion, we recommend that members of the IAGA V-MOD working group consider revising the construction of IGRF to enhance the model’s utility. Specifically, we suggest evaluating whether the IGRF-15 should evolve to include $l_{SV}=9-13$ to better align with the MF model and reflect advancements in global geomagnetic observation capabilities.

## Figures and Tables

**Fig. 7:**
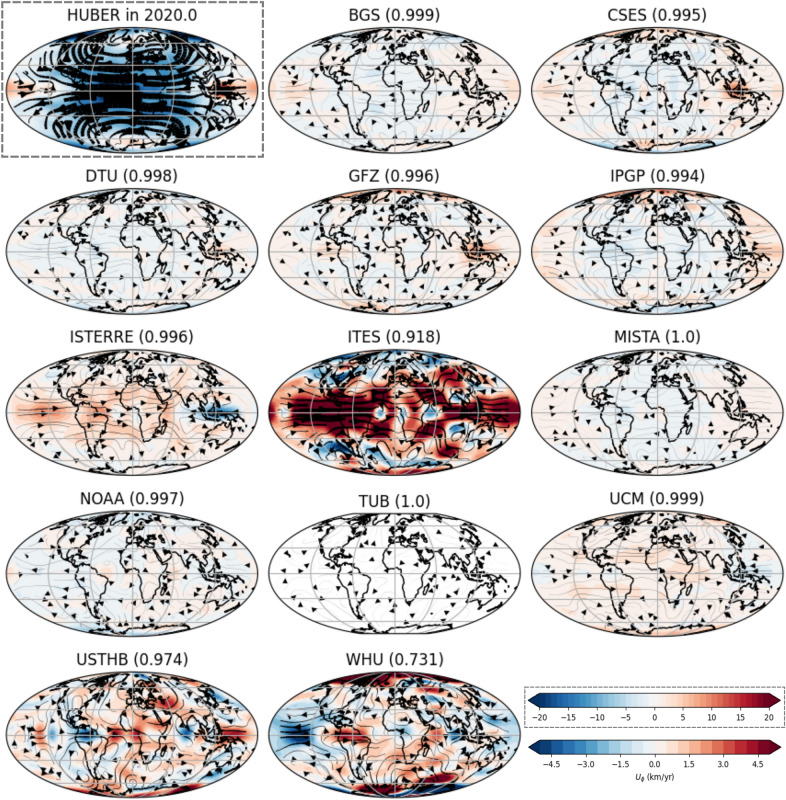
Predicted flow for the 2020.0 timestep for the published Huber-weighted mean model in space IGRF model (shown within grey dashed box) along with the difference between each candidate and the published IGRF model. The number in the brackets after each candidate name indicates the correlation coefficient between the $U_\phi$ of the flow model inverted from the Huber-weighted mean model in space and $U_\phi$ of the flow model of the candidate. The colorbar for all models are consistent and shown for in the bottom right corner, except for the weighted mean model in space which is shown in its own grey dashed box

**Fig. 8:**
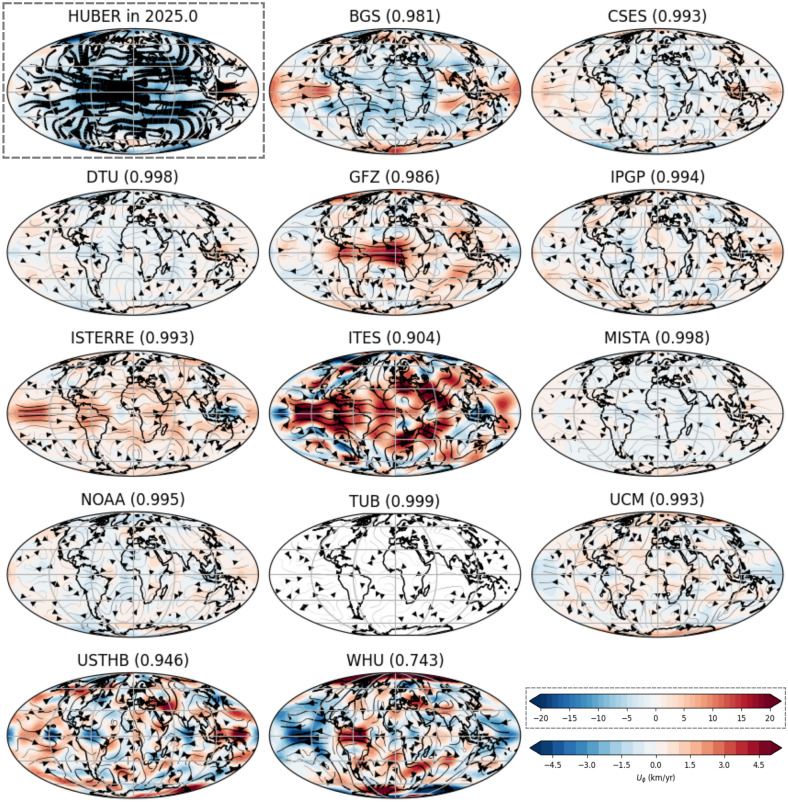
As with Fig. 7 but for epoch 2025.0. The colorbar for all models are consistent and shown for in the bottom right corner, except for the weighted mean model in space which is shown in its own grey dashed box

## Data Availability

The data sets used during the current study and plots of candidate comparisons are available from the IAGA Working Group V-MOD GitHub for the evaluation of IGRF-14 coefficients: https://github.com/IAGA-VMOD/IGRF14eval . Further information the work of the International Association of Geomagnetism and Aeronomy (IAGA) can be found on their website: https://iaga-aiga.org/ . Further information on the IAGA Working Group V-MOD, which oversees the IGRF construction, can be found on their website: https://www.ncei.noaa.gov/services/world-data-system/v-mod-working-group . Swarm and Cryosat-2 data are available from: https://earth.esa.int/web/guest/swarm/data-access . CHAMP data can be obtained from: http://isdc.gfz-potsdam.de . Ørsted and SAC-C data are available from: https://www.space.dtu.dk/english/research/scientific_data_and_models/magnetic_satellites . CSES data are available from: http://www.leos.ac.cn . Macau Science Satellite data are available from: https://mss.must.edu.mo/ . INTERMAGNET data are available from: https://www.intermagnet.org . The full Kalmag model (TUB candidate) is available at: https://ionocovar.agnld.uni-potsdam.de/Kalmag/Kalmag/Model/ . The full CHAOS-8 model (DTU) is available at: https://www.spacecenter.dk/files/magnetic-models/CHAOS-8/ . The pygeodyn core flow inversion documentation (including instructions for installation) is available at: https://geodynamo.gricad-pages.univ-grenoble-alpes.fr/pygeodyn/index.html. The core flow inversion models and Jupyter notebook for plotting will be accessible at 10.5281/zenodo.17494071 .

## Appendix 1: Flow maps in 2020.0 and 2025.0

Figures 7 and 8 show comparisons of flow model inversions in 2020.0 (the DGRF epoch) and 2025.0 (the IGRF epoch) in a similar style to Fig. 3. The number in brackets after each candidate name is the correlation coefficient between the $U_\phi$ of the flow model inverted from the Huber-weighted mean model in space and $U_\phi$ of the flow model of the candidate.

## Abbreviations

$a$: Earth’s reference radius
AR: Auto-regressive ASM Absolute Scalar Magnetometer
$\textbf{B}$: Vector of magnetic main field
$B_r$: Radial magnetic main field
$c$: Radius of core surface
CMB: Core-Mantle Boundary
CSES: China Seismo-Electromagnetic Satellite
DGRF: Definitive Geomagnetic Reference Field
ER: Errors of Representativeness (unmodelled diffusion and small-scale flow)
$g^m_l$ and $h^m_l$: Schmidt semi-normalized Gauss coefficient for main field
$\dot{g}^m_l$ and $\dot{h}^m_l$: Schmidt semi-normalized Gauss coefficient for SV
IAGA: International Association of Geomagnetism and Aeronomy
IGRF: International Geomagnetic Reference Field (number corresponds to particular generation)
$L$: Maximum spherical harmonic degree
$l$: Spherical harmonic degree
$m$: Spherical harmonic order
MF: Main field
MSS-1: Macau Science Satellite 1
$r$: Radius of model
*SV*: Secular variation, first time-derivative of the magnetic field
U: Core surface flow
$\textbf{u}_h$: Horizontal core motion VFM Vector Field Magnetometer
$$\theta$$: Colatitude
$$\eta$$: Magnetic diffusivity
$$\phi$$: Longitude
